# Abdominal sacrocolpopexy: could we simplify the technique?

**DOI:** 10.61622/rbgo/2025rbgo74

**Published:** 2025-11-18

**Authors:** Edilson Benedito de Castro, Andressa Soares Castro Alves, Luiz Gustavo de Oliveira Brito, Glaucia Miranda Varella Pereira, Juliana do Carmo Fazzolari, Cássia Raquel Teatin Juliato

**Affiliations:** 1 Faculdade de Ciências Médicas da Universidade Estadual de Campinas Departmentamento de Obstetrícia e Ginecologia Campinas SP Brasil Departmentamento de Obstetrícia e Ginecologia, Faculdade de Ciências Médicas da Universidade Estadual de Campinas, Campinas, SP, Brasil.

**Keywords:** Apical genital prolapse, Sacrocolpopexy, Pelvic organ prolapse, Uterine prolapse, Sutures, Urogenital surgical procedures

## Abstract

**Objective:**

To compare the efficacy of a traditional open ASC technique (ASC-T) to an open modified technique (ASC-M).

**Methods:**

Retrospective cohort study with stage 3 or 4 apical prolapse women, who operated on using one of the two techniques were included in the study: ASC-T (vaginal mesh is secured with eight sutures) and ASC-M (four sutures). The POP-Q was used to objectively assess anatomical improvement. Women with less than one year of follow-up, without POP-Q classification, or with incomplete data were excluded.

**Results:**

A total of 223 women underwent ASC: 120 in ASC-T and 103 in ASC-M. The average age was 65.3 (±6.5) years in the ASC-T group and 65 (±8.5) years in the ASC-M group, with no difference between them (p=0.706). There was no difference in intraoperative increased bleeding (p=1.000) and bladder injury (p=0.706) in both groups. Comparing the POP-Q points pre- and postoperatively, we observed improvement in all points in both groups (p<0.001) without difference between them. The analysis of variance for repeated measures was used to compare the outcomes between the two groups. The postoperative prolapse stage was similar between the two groups in the apical (p=0.251) and anterior (p=0.052) vaginal compartments. In the subjective evaluation, we observed a high rate of cure and improvement in both groups, respectively 81.7% and 16.7% in the ASC-T group, and 91.3% and 8.7% in the ASC-M group (p=0.100).

**Conclusion:**

Both sacrocolpopexy techniques were effective in treating apical prolapse, as evidenced by both objective and subjective cure rates, with a low complication rate.

## Introduction

Pelvic organ prolapse (POP) is characterized by the descent of one or more pelvic structures, including the anterior or posterior vaginal walls, the uterus (or cervix), or the vaginal apex (the vaginal vault after a hysterectomy). The diagnosis of POP is clinically significant only when these anatomical changes are accompanied by symptoms, most commonly manifesting at or beyond the hymenal level.^(1,2)^

In a large-scale study conducted by the Women's Health Initiative (WHI) in the U.S., involving 16,616 women aged 50 to 79 with an intact uterus, the prevalence of pelvic organ prolapse was substantial: 34.3% had cystocele, 14.2% had uterine prolapse, and 18.6% had rectocele.^(3)^ Several risk factors are associated with the development of POP, including vaginal childbirth, higher parity, infant birth weight, advanced age, increased body mass index (BMI), levator ani muscle defects, and a larger hiatal area.^(4)^ Additional risk factors for surgical recurrence include levator avulsion, stage 3 or 4 POP, previous prolapse surgery, and a larger genital hiatus during the Valsalva maneuver.^(5)^ Notably, anterior vaginal wall prolapse is strongly associated with apical prolapse, being present in 42% of stage 2 cystoceles, 85% of stage 3, and 100% of stage 4 cystoceles.^(6)^

Among the surgical options for apical prolapse, abdominal sacrocolpopexy (ASC) has demonstrated superior outcomes in terms of both subjective and objective success rates, lower reoperation rates due to recurrence, and reduced incidence of stress urinary incontinence compared to vaginal surgical techniques. As a result, ASC is widely regarded as the gold standard for apical prolapse repair, despite its principal drawback of longer operative time.^(7)^ The data were supportive of equivalence in anatomical outcomes in all access routes for sacral colpopexy, although perioperative outcomes support some benefits of laparoscopic access as compared to both open and robotic interventions.^(7)^ However, there is no standardized approach to performing ASC,^(8)^ leading to significant variation in surgical techniques, but in most of them, eight suture points are made to fix the mesh to the vaginal wall and two to secure the mesh to the anterior longitudinal ligament of the sacral promontory. This study aims to compare a traditional ASC technique (ASC-T), in which the vaginal mesh is affixed with eight sutures, with a modified version (ASC-M) that employs four sutures. The modification intends to reduce the extent of vaginal dissection, thereby simplifying the procedure and decreasing operative time.

## Methods

A retrospective cohort study, conducted at the Pelvic Medicine and Reconstructive Surgery Sector of the Department of Obstetrics and Gynecology at the University of Campinas, included women with apical prolapse who underwent open ASC-T or open ASC-M. The STROBE checklist was used as a guide for this study.

For participant inclusion, a database was used that includes all the data of women with genital prolapse who underwent surgery at the service between 2003 to 2022. Women with stage 3 or 4 apical prolapse, classified according to POP-Q,^(2)^ and who were operated on using one of the two techniques were included in the study. Women with less than one year of follow-up, without POP-Q classification, or with incomplete data were excluded.

All surgeries were performed or supervised by the same surgeon, who has experience in reconstructive pelvic floor surgery. The ASC-T surgeries were performed until 2017, after which only ASC-M surgeries were conducted. All surgeries were open abdominal sacrocolpopexy.

Socio-demographic, obstetric, gynecological data, physical examination findings, urinary symptoms, surgical history, previous comorbidities, and intra- and postoperative complications were evaluated. The primary outcome was the post-operative prolapse, classified according to POP-Q, and self-reported by the women through four options: cured, improved, unchanged, and worse. Intraoperative complications were also evaluated, such as reports of increased bleeding, evaluated through the perception of the surgical team and organ injuries, including bladder or bowel damage. The postoperative complications assessed included vaginal mesh erosion, presence of urinary incontinence or de novo urinary incontinence, urgency or urge incontinence, and nocturia.^(1)^

In all surgeries, macroporous polypropylene type 1 mesh was used. In the traditional surgery group (ASC-T), a generic mesh was trimmed into a 15x5 cm shape for each vaginal compartment (anterior and posterior). In the modified surgery group (ASC-M), a Y-shaped mesh (UPSYLON BOSTON) was utilized. In both groups, the mesh was attached to the vagina and the anterior longitudinal ligament of the sacral promontory using 0 polypropylene non-absorbable suture. All patients received prophylactic antibiotic coverage with three doses of cefazolin (2g+2g+2g) and three doses of metronidazole (1g+500mg+500mg), except for those allergic, where clindamycin (900mg+600mg+600mg) was used instead.

The procedure begins with opening the retroperitoneal sacral space, extending from the promontory to the posterior cul-de-sac, exposing the anterior longitudinal ligament of the sacral promontory. The retovaginal space is dissected up to the upper two-thirds of the vagina, followed by dissection of the vesicovaginal space to the level of the vesical neck. A supracervical hysterectomy is performed in women with a uterus.

The mesh is secured with 0 polypropylene sutures. The posterior mesh is fixed with two sutures to the most distal part of the dissected posterior vaginal wall and two sutures to the vaginal apex or posterior cervix. The anterior mesh is fixed with two sutures to the anterior vaginal apex or cervix and two sutures to the vaginal wall at the level of the vesical neck. The superior edge of the mesh is anchored to the anterior longitudinal ligament of the sacral promontory with two sutures. The mesh is then peritonealized, and a perineorrhaphy is performed when necessary.

**Figure 1:**
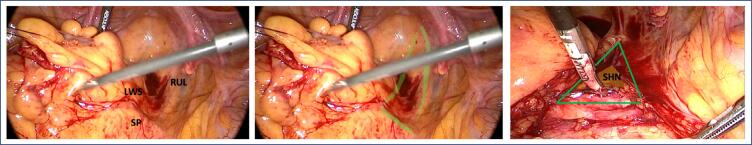
First pyramid: sacral retroperitoneal dissection. Illustrative laparoscopic view only

**Figure 2:**
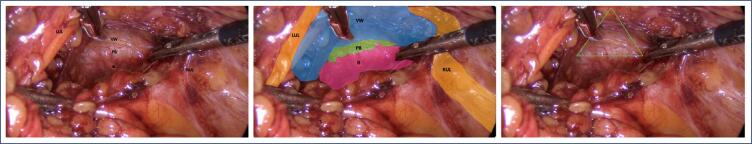
Second pyramid: posterior vaginal wall dissection. Illustrative laparoscopic view only

**Figure 3:**
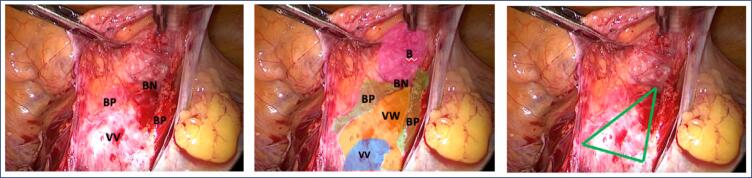
Third pyramid: anterior vaginal wall dissection. Illustrative laparoscopic view only

In this technique, referred to as the "Pyramids of Giza Technique," three key dissections are performed using anatomical structures resembling the silhouette of pyramids. These dissections occur within a triangular area:

- Sacral Retroperitoneal Dissection (Figure 1): The base of the pyramid is the sacral promontory, the lateral-medial wall is the right lateral wall of the sigmoid colon, and the lateral-lateral wall is the right uterosacral ligament. It's important to note that the superior hypogastric nerve crosses the central area of this pyramid.
- Posterior Vaginal Wall Dissection (Figure 2): The superolateral walls of the pyramid are the uterosacral ligaments, and the inferolateral walls are the lateral wall of the rectum. The base of the pyramid is the perineal body.
- Anterior Vaginal Wall Dissection (Figure 3): The base of this pyramid is the cervix or vaginal apex, and the lateral walls are the vesical pillars up to the vesical neck.

SP - sacral promontory; LWS - right lateral wall of the sigmoid; RUL - right uterosacral ligament; SHN - superior hypogastric nerve

VW - vaginal wall; PB - perineal body; R - rectum; RUL - right uterosacral ligament; LUL - left uterosacral ligament; S - sigmoid colon

VV - vaginal vault; BP - bladder pillar; BN - bladder neck; VW - vaginal wall; B: bladder

In women with a uterus, a supracervical hysterectomy is performed. The mesh is secured with 0 polypropylene sutures. The posterior mesh is anchored with one suture to the perineal body and one suture to the vaginal apex or posterior cervix. The anterior mesh is secured with one suture to the anterior vaginal apex or cervix and one suture to the vaginal wall at the vesical neck level. The superior edge of the mesh is anchored to the anterior longitudinal ligament of the sacral promontory with a single double suture. The mesh is then peritonealized, and perineorrhaphy is performed when necessary.

For an 80% study power and a 5% significance level, a sampling of 45 patients was needed to detect a 25% difference between surgical routes. Descriptive analysis was performed to characterize the sample according to study variables. For categorical variables, frequency distributions were presented as absolute values (n) and corresponding percentages (%). For continuous variables, descriptive statistics included the mean, standard deviation, minimum and maximum values, median, and interquartile range.

To assess differences in categorical variables between groups, the Chi-square test was employed, or Fisher's exact test was used when expected cell counts were less than five. Given the non-normal distribution of numerical variables, the Mann-Whitney U test was used for between-group comparisons.

For paired categorical data, the McNemar test was applied when variables had two categories, and Bowker's test of symmetry was used for variables with three or more categories. The Wilcoxon signed-rank test was applied to compare paired numerical variables between time points, again due to non-normality.

To evaluate numerical variables across both groups and time points simultaneously, repeated measures analysis of variance (ANOVA) was conducted. When significant effects were found, post hoc analyses were performed using Tukey's test for between-group comparisons and profile contrast tests for within-group differences over time. Variables that did not meet normality assumptions were rank-transformed prior to analysis.

A two-sided significance level of 5% (P < 0.05) was adopted for all statistical tests.

The study was approved by the hospital's research committee and the ethics committee of the State University of Campinas 6.724.590 (Certificado de Apresentação para Apreciação Ética – CAAE: 77220023.8.0000.5404).

## Results

A total of 223 women underwent ASC between 2003 and 2022: 120 in the traditional surgery group (ASC-T) and 103 in the modified surgery group (ASC-M), with follow-up of 623(±337) days in the ASC-T group and 543(±283) days in the ASC-M group, with no significant difference between them (p=0.189). The average age was 65.3(±6.5) years in the ASC-T group and 65(±8.5) years in the ASC-M group, with no significant difference between them (p=0.706). Parity and the number of vaginal deliveries were higher in the ASC-T group (3.91 ±2.84 and 3.48 ±2.62) compared to the ASC-M group (2.69 ±1.46 and 2.39 ±1.13), with statistical significance (p=0.001 and 0.005, respectively). There was no difference in menopausal status, presence of comorbidities, history of previous surgeries, or urinary symptoms between the two groups (Table 1).

**Table 1:** Baseline Characteristics Between Groups

Patient demographics		ASC-T (n=120)	ASC-M (n=103)	p-value
Age (years)		65.3(±6.5)	65(±8.5)	0.706*
Parity		3.91(±2.84)	2.69(±1.46)	0.001*
Vaginal delivery		3.48(±2.62)	2.39(±1.13)	0.005*
Menopouse		116(96.67)	97(94.17)	0.519**
Arterial hypertension		65(54.17)	50(48.54)	0.402***
Diabetes		13(10.83)	13(12.62)	0.678***
Previous surgery				
	Hysterectomy	34(28.33)	27(26.21)	0.723***
	Sacrocolpopexy	2(1.67)	3(2.91)	0.664**
	High MCCALL	1(0.83)	2(1.94)	0.597**
	Sacrospinous fixation	3(2.50)	4(3.88)	0.706**
	Anterior colporrhaphy	45(37.50)	30(29.13)	0.187***
	Posterior colporrhaphy	45(37.50)	32(31.07)	0.314***
	SLING	15(12.50)	9(8.74)	0.366***
Urinary symptoms				
	Stress urinary incontinence N (%)	24(20)	19(18.45)	0.769***
	Urgency N (%)	53(44.17)	43(41.75)	0.716***
	Urge incontinence N (%)	45(37.50)	30(29.13)	0.187***
	Nocturia N (%)	34(28.33)	31(30.10)	0.773***
POP-Q (cm)				
	Aa	+1.01(-3 to +3)	-0.43(-3 to +3)	<0.001*
	Ba	+4.79(-1 to +11	+4.17(0 to +9)	0.037*
	C	+4.30(+2 to +11).	+3.63(+2 to +9)	0.068*
	GH	2.49(1 to 8).	2.16(1 to 4).	0.141*
	PB	2.56(1 to 4).	2.76(1 to 4).	0.065*
	TVL	8.17(5 to 11)	8.10(7 to 9)	0.661*
	Ap	-1.48(-3 to +3)	-1.52(-3 to +3)	0.648*
	Bp	+2.60(-3 to +10	+1.87(-3 to +8)	0.140*
	D	+1.88(-3 to +10)	+1.03(-3 to +8)	0.163*
POP-Q Stage N (%)				
	Apical compartment			0.018***
	3	84(70)	86(83.50)	
	4	36(30)	17(16.50)	
	Anterior compartment			0.037**
	2	1(0.83)	1(0.97)	
	3	83(69.17)	85(82.52)	
	4	36(30)	17(16.50)	
	Posterior compartment			0.167**
	0	4(3.33)	4(3.88)	
	1	1(0.83)	2(1.94)	
	2	43(35.83)	46(44.66)	
	3	36(30)	34(33.01)	
	4	36(30)	17(16.50)	

ASC-T traditional surgery group; ASC-M modified surgery group; POP-Q Pelvic Organ Prolapse Quantification;

* Mann-Whitney Test;

** Fisher's exact test;

*** chi-square test

Regarding the POP-Q points, there was no significant difference in the average point C, which represents the apex, between the two groups (p=0.068). However, participants in the ASC-T group had higher stage of prolapse for anterior vaginal wall points (Aa and Ba) compared to those in the ASC-M group (p<0.001 and 0.037). No significant differences were observed in other POP-Q stage points. Furthermore, a higher proportion of women in the ASC-M group were classified as stage 3 in comparison to those in the ASC-T group (p=0.018) (Table 1).

There was no significant difference in the number of sling surgeries performed concomitantly with ASC between the two groups (p=0.960). However, more women in the ASC-T group underwent perineorrhaphy (p=0.014). When comparing intraoperative complications, there were 2 cases of increased bleeding in each group (p=1.000), and 3 cases of bladder injury in the ASC-T group versus 4 in the ASC-M group (p=0.706). No cases of bowel injury were reported in either group. The rate of late postoperative pain (1 year) was 13.3% in the ASC-T group and 5.8% in the ASC-M group (p=0.061). There was only one case of minor vaginal mesh erosion in the anterior vaginal wall, which occurred in the ASC-T group (p=1.000).

The incidence of postoperative urinary symptoms was similar between the groups: stress urinary incontinence (p=0.146), urgency (p=0.098), urge incontinence (p=0.308), nocturia (p=0.309), de novo stress urinary incontinence (p=0.056), and de novo urge incontinence (p=0.309) (Table 2).

**Table 2:** Intra- and postoperative outcomes

Procedure outcomes		ASC-T (n=120)	ASC-M (n=103)	p-value
Follow-up (days)		623.68(±337.82)	543.70(±286.15)	0.189*
Concomitant procedures				
	Perineorrhaphy	49(40.83)	26(25.24)	0.014**
	SLING	6(5)	5(4.85)	0.960**
	Delayed SLING (after ASC)	5(4.17)	7(6.80)	0.386**
Intraoperative complications				
	Increased bleeding	2(1.67)	2(1.94)	1.000***
	Bladder injury	3(2.50)	4(3.88)	0.706***
Postoperative complications				
	Pain	16(13.33)	6(5.83)	0.061**
	Mesh erosion	1(0.83)	0(0)	1.000***
Urinary symptoms				
	Stress urinary incontinence	28(23.33)	33(32.04)	0.146**
	Urgency	28(23.33)	15(14.56)	0.098**
	Urge incontinence	25(20.83)	16(15.53)	0.308**
	Nocturia	17(14.17)	10(9,71)	0.309**
	Stress urinary incontinence	19(15.83)	27(26.21)	0.056**
	Urge incontinence	17(14.17)	19(9.71)	0.309**
POP-Q Stage N (%)				
	Apical compartment			0.251***
	0	114(95)	103(100)	
	1	3(2.5)	0(0)	
	2	1(0.83)	0(0)	
	3	1(0.83)	0(0)	
	4	1(0.83)	0(0)	
	Anterior compartment			0.052***
	0	90(75)	92(89.32)	
	1	2(1.67)	1(0.97)	
	2	23(19.17)	9(8.74)	
	3	4(3.33)	1(0.97)	
	4	1(0.83)	0(0)	
	Posterior compartment			<0.001***
	0	79(65.83)	90(87.38)	
	1	3(2.50)	0(0)	
	2	34(28.33)	12(11.65)	
	3	3(2.50)	1(0.97)	
	4	1(0.83)	0(0)	
Subjective cure N (%)				0.100***
Cure		98(81.67)	94(91.26)	
Improvement		20(16.67)	9(8.74)	
Unchanged		1(0.83)	0(0)	
Worse		1(0.83)	0(0)	

ASC-T traditional surgery group; ASC-M modified surgery group; POP-Q Pelvic Organ Prolapse Quantification;

* Mann-Whitney Test;

** chi-square test;

*** Fisher's exact test

The postoperative prolapse stage was similar between the two groups in the apical vaginal compartment (p=0.251). However, in the posterior (p<0.001) and anterior (p=0.052) vaginal compartments, we found a higher rate of stage 0 prolapse in the ASC-M group and stage 2 prolapse in the ASC-T group. In the subjective evaluation, we observed a high rate of cure and improvement in both groups, respectively 81.7% and 16.7% in the ASC-T group, and 91.3% and 8.7% in the ASC-M group (p=0.100) (Table 2).

Comparing the POP-Q points pre- and postoperatively, we observed improvement in all points in both groups (p<0.001), except for TVL in the ASC-T group, which did not show a significant increase (p=0.242). Using ANOVA (analysis of variance) to assess the difference between the pre- and postoperative values between the groups, we found greater improvement in the ASC-M group compared to the ASC-T group in the TVL (p<0.001) and Ap points (p=0.007). There was no significant difference between the groups in the Aa (p=0.100), Ba (p=0.584), C (p=0.085), GH (p=0.571), PB (p=0.497), Bp (p=0.068), and D points (p=0.465) (Table 3).

**Table 3:** Pelvic Organ Prolapse Quantification System score before and 1 year after surgery

Variable*	ASC-T (n=120)			ASC-M (n=103)			Mean difference**	
POP-Q	Preop	Postop	p-value***	Preop	Postop	p- value***	Mean	p- value****
Aa	1.01 ±2.28	-2.23 ±1.27	<0.001	-0.43 ±1.83	-2.64 ±0.87	<0.001	1.02 ±2.03	0.100
Ba	4.79 ±2.30	-2.04 ±1.70	<0.001	4.17 ±1.89	-2.63 ±0.90	<0.001	0.03 ±2.05	0.584
C	4.30 ±2.43	-7.56 ±2.06	<0.001	3.63 ±1.93	-8.30 ±0.57	<0.001	-0.07 ±2.55	0.085
GH	2.49 ±1.19	1.75 ±0.72	<0.001	2.16 ±0.77	1.65 ±0.47	<0.001	0.22 ±0.94	0.571
PB	2.56 ±0.76	3.01 ±0.65	<0.001	2.76 ±0.62	3.09 ±0.49	<0.001	-0.12 ±0.79	0.497
TVL	8.17 ±0.95	8.17 ±1.73	0.242	8.10 ±0.53	8.53 ±0.57	<0.001	0.43 ±1.26	<0.001
Ap	-1.48 ±1.92	-2.11 ±1.32	<0.001	-1.52 ±1.70	-2.61 ±0.83	<0.001	-0.45 ±1.83	0.007
Bp	2.60 ±3.21	-1.86 ±1.72	<0.001	1.87 ±2.58	-2.59 ±0.91	<0.001	-0.01 ±3.07	0.068
D	2.23 ±3.41	-7.73 ±1.90	<0.001	1.04 ±2.45	-8.33 ±2.04	<0.001	0.59 ±3.28	0.465

ASC-T traditional surgery group; ASC-M modified surgery group; POP-Q Pelvic Organ Prolapse Quantification;

* Wilcoxon Test;

** ANOVA for repeated measures;

*** Mean ± standard deviation;

**** Difference between subtraction of pre- and post-values between groups

## Discussion

The findings of this study showed that ASC is highly effective in improving apical prolapse, with no difference between the ASC-T and the ASC-M regarding the objective parameters evaluated by POP-Q and the subjective outcomes reported by the participants. Additionally, there were no differences in complication rates.

ASC is considered the gold standard surgery for the treatment of uterine and vaginal vault prolapse.^(7)^ The present findings suggest that both ASC-T and ASC-M are effective in the surgical management of stage 3 and 4 apical prolapse, as classified by the POP-Q system, achieving high anatomical success rates at point C. The observed improvements in pre- and postoperative values across both groups are consistent with previously published literature.^(9–13)^

However, ASC-M offers certain advantages, as it utilizes fewer sutures in the vagina, potentially lowering the risk of mesh erosion and dyspareunia. In our study, though, the occurrence of vaginal mesh erosion was minimal (with only one case reported in the traditional technique), limiting our ability to definitively confirm this hypothesis. Additionally, the modified technique requires less dissection and results in shorter surgical times without compromising effectiveness.

A higher incidence of stage 2 anterior vaginal wall prolapse was observed postoperatively in the ASC-T group, whereas a greater proportion of stage 0 prolapse was noted in the ASC-M group. This finding is likely attributable to the higher prevalence of stage 4 anterior compartment prolapse in the ASC-T group at preoperative period, which may have influenced postoperative anatomical outcomes.

Superior postoperative outcomes in the posterior compartment were observed in the ASC-M group, which may be attributed to differences in the surgical approach between the two techniques. In ASC-T, the mesh is dissected and anchored to the upper two-thirds of the posterior vaginal wall. In contrast, ASC-M involves a deeper dissection extending to the perineal body, where the mesh is secured with just one suture. Despite using only a single fixation point, this more extensive dissection provided a more effective correction of the posterior prolapse. More women in the ASC-T group underwent perineorrhaphy (p=0.014), which did not result in improved anatomical outcomes compared to the ASC-M group. A study comparing perineorrhaphy with no perineorrhaphy during sacrocolpopexy showed no difference in the improvement of anatomical outcomes in the posterior vaginal compartment between the two groups.^(14)^ We believe that perineorrhaphy should be performed in specific cases, based on measurements of GH, PB, perineal muscle strength, and the woman's complaint of vaginal laxity.

Regarding vaginal length, we observed no change in TVL in the ASC-T group, but there was a significant increase (0.43cm) in the ASC-M group. We believe that the lower number of sutures in ASC-M, four compared to eight in ASC-T, resulted in less fibrotic reaction to the mesh^(15–17)^ and, consequently, less vaginal retraction. This likely contributed to the improvement in TVL in the ASC-M group.

Despite the improvement in some objective variables in the ASC-M group compared to the ASC-T group, we did not observe any difference in subjective cure rates, with both techniques providing a high rate of improvement. A recent publication advocates that the assessment of cure should not be solely anatomical but should focus primarily on the interaction between anatomical cure and subjective cure.^(18)^

By using fewer sutures to attach the mesh to the vagina, we can perform less extensive dissections. Additionally, by employing what we call the "Pyramids of Giza Technique", we can use well-defined anatomical reference points during dissections, potentially making the surgery easier to perform, with theoretically lower risk of complications. However, despite these theoretical benefits, the rates of bleeding and bladder injury were the same in both groups, and there were no cases of bowel injury.

Although both meshes used in the surgeries were made of macroporous polypropylene type 1, they were from different manufacturers. In the ASC-T surgery group, we used a generic mesh, and in the ASC-M surgery group, we used a Y-shaped mesh (UPSYLON BOSTON). This does not seem to have influenced the results, as the rates of postoperative pain and vaginal erosion were similar.

One of the strengths of this study lies in the introduction of an innovative surgical technique that simplifies the current gold standard approach traditionally described in the literature. Additionally, the inclusion of a substantial sample of women from a clinically homogeneous population strengthens the study findings. However, the retrospective design of the study represents a limitation, as it inherently introduces potential bias.

While the cure rates for abdominal, laparoscopic, and robotic sacrocolpopexy are comparable, laparoscopic surgery has a longer operative time but less bleeding and better recovery than the abdominal approach. Compared to the robotic approach, laparoscopic surgery offers shorter operative times, lower costs, and less immediate postoperative pain.^(7,19)^ Another limitation of this study was the use of only the open abdominal approach to compare the two techniques, rather than a minimally invasive approach such as laparoscopy, which is considered the gold standard. Based on the findings of this study, we advocate for further research comparing prospectively the traditional technique with the modified technique in minimally invasive sacrocolpopexy.

## Conclusion

In conclusion, both sacrocolpopexy techniques proved effective in the treatment of apical prolapse, demonstrating favorable objective and subjective cure rates, along with a low incidence of complications. Notably, mesh fixation extending to the perineal body was associated with improved correction of posterior compartment prolapse when compared to fixation limited to the lower two-thirds of the vaginal wall.
